# A Microtitre Plate Dilution Method for Minimum Killing Concentration Is Developed to Evaluate Metabolites-Enabled Killing of Bacteria by β-lactam Antibiotics

**DOI:** 10.3389/fmolb.2022.878651

**Published:** 2022-06-27

**Authors:** Jian-jun Tao, Juan-juan Xiang, Ming Jiang, Su-fang Kuang, Xuan-xian Peng, Hui Li

**Affiliations:** ^1^ State Key Laboratory of Bio-Control, School of Life Sciences, Southern Marine Science and Engineering Guangdong Laboratory (Zhuhai), Sun Yat-sen University, Guangzhou, China; ^2^ Laboratory for Marine Fisheries Science and Food Production Processes, Qingdao National Laboratory for Marine Science and Technology, Qingdao, China

**Keywords:** antibiotic resistance, minimum killing concentration, microtitre plate dilution method, metabolite, reprogramming metabolomics, inosine, bacteria

## Abstract

Because, as of yet, there are few new antibiotics active against multidrug-resistant bacteria are being explored, compounds including metabolites that might help us tide over this crisis are greatly expected. A recently adopted method to evaluate the potentiation of metabolites is the plate-counting test. However, the method is time-consuming, strenuous, and unfeasible for a large scale of screening. A minimum inhibitory concentration (MIC) test by using a microtitre plate dilution method is convenient and economic for a large scale of identification, but it cannot be used to detect the potentiation. Here, the microtitre plate dilution method was modified to develop a novel test for evaluating metabolites that enable the killing of bacterial pathogens by antibiotics, designed as minimum killing concentration (MKC). To do this, bacterial number, incubation time, ionic strength of M9 medium, and inosine concentration are optimized using *Escherichia coli*. Different from the MIC test, which uses 5 × 10^4^ CFU cells and performed in LB medium, the MKC test needed 1 × 10^7^ CFU - 2 × 10^7^ CFU cells and was carried out in M9 medium. Moreover, MKC test was suitable for bactericidal antibiotics such as cephalosporins, penicillins and carbapenems and was proportional to the plate-counting test. The developed MKC test was feasible for different metabolites and clinically multidrug-resistant pathogens, and measurement of minimum bactericidal concentration (MBC). Therefore, the MKC test was developed to accelerate the identification of compounds that promote antibiotic-mediated killing efficacy.

## Introduction

The wide spread of antibiotic-resistant bacteria is a growing problem, imposing a catastrophic threat to humans and the animal industry throughout the world. The invention of new antibiotics and the potentiation of the current used antibiotics are two major measures to cope with the antibiotic-resistant pathogens. Because, as of yet, few new antibiotics active against multidrug-resistant bacteria are being explored, compounds including metabolites that might help us tide over this crisis are greatly expected.

Recently, it has been documented that the metabolic environment modulates antibiotic-mediated killing efficacy (Allison et al., 2011; Peng et al., 2015a; Peng et al., 2015b). Using reprogramming metabolomics, key biomarkers are identified by comparison between antibiotic-sensitive and -resistant bacteria and then the identified key biomarkers are used to potentiate antibiotic-mediated killing efficacy (Su et al., 2018; Cheng et al., 2019; Li et al., 2020; Kuang et al., 2021a; Zhao et al., 2021). The approach has resulted in the identification of several metabolites including glucose, alanine, pyruvate, and glutamine/inosine as crucial biomarkers that elevate antibiotic uptake to promote the antibiotic-mediated killing (Peng et al., 2015a; Su et al., 2018; Zhang et al., 2019; Li et al., 2020; Zhao et al., 2021). During the identification, evaluation on metabolite capability in the metabolite-enabled killing by antibiotics is especially key. A recently adopted method to the evaluation is the plate-counting test (Peng et al., 2015b; Kuang et al., 2021a; Kuang et al., 2021b; Su et al., 2021). The method is accurate, but time-consuming, strenuous, and unfeasible for a large scale of screening. Therefore, the development of new approaches is necessary for identification of metabolites that potentiate antibiotic-mediated killing.

A minimum inhibitory concentration (MIC) test determines the antimicrobial activity of a material against a specific bacterium. Now, the most commonly employed way is microtitre plate dilution method. The method is convenient, rapid, and economic, especially for a large scale of screening. However, it is performed in LB medium, which cannot guarantee that the metabolites evaluated are utilized since LB medium is a rich nutrient medium. Therefore, development of a similar approach such as an MIC test based on microtitre plate dilution method (MIC test) is required for the identification of metabolites that promote antibiotic-mediated killing and for the examination of the experimental procedures. Further optimization for procedures will benefit the use of the test.

Cefperazone-sulbactam (SCF) is a cephalosporin antibiotic widely used in clinic and SCF-resistant bacteria is frequently isolated (Li et al., 2018; Huai et al., 2019; Guclu et al., 2020). On the other hand, depressed inosine was documented in antibiotic-resistant bacteria, suggesting that inosine may be a potential metabolite that increases SCF-mediated killing (Zhao et al., 2021). Here, we utilized SCF and inosine to develop a microtitre plate dilution method to determine metabolite-enabled killing efficacy. Comparatively, MIC is the smallest amount of a compound that limits visible microbial growth in culture, whereas the metabolite-enabled killing by antibiotic is the smallest amount of a compound that kills visible microbial growth in culture, it is thereby designed as minimum killing concentration (MKC). The present study focuses on the optimization for the procedures of MKC.

## Materials and Methods

### Bacterial strains and culture conditions

Bacterial *E. coli* strains used in this study were from the collection of our laboratory. These strains were grown at 37°C in 50 ml LB (1% bacterial peptone, 0.5% yeast extract, and 1% NaCl) overnight and collected by centrifugation at 8000 × rpm for 3 min. Antimicrobial agents including cefperazone-sulbactam, cefperazone, ceftriaxone, ceftazidime, cefotaxime, cefradine, ampicillin, and meropenem were purchased from a commercial source (Shanghai Sangon Biological Engineering Technology & Services Co. Ltd., China).

### MIC determination

MIC was determined by antimicrobial susceptibility testing according to CLSI guidelines (Delgado-Valverde et al., 2020; Zhang et al., 2020). In brief, overnight bacteria cultured in LB medium were diluted at 1:100 in fresh LB and cultured at 37°C with shaking at 200 rpm to an optical density at 600 nm of 0.5. Then, 10 μL of 5 × 10^4^ CFU was added into each well of a 96-well microtiter polystyrene tray with 100 μL LB of a series of 2-fold dilutions of an antibiotic. The mixtures were incubated at 37°C for 16 h. MIC was defined as the lowest antibiotic concentration that inhibited visible bacteria growth.

### MKC determination

Overnight bacteria cultured in LB medium were collected and washed three times with saline solution before being suspended in M9 medium (17.1 g Na_2_HPO_4_.12H_2_O, 3 g KH_2_PO_4_, 1g NH_4_Cl, 0.5 g NaCl, 0.82 g CH_3_COONa, 0.24 g MgSO4, 0.0111 g CaCl_2_ dissolved in 1 L water) to an optical density at 600 nm of 1.0, and then diluted at 1: 8 or 1:16 with M9 medium. Aliquot of 100 μL of 1×10^7^ or 2 × 10^7^ CFU was added into each well of a 96-well microtiter polystyrene tray with 100 μL M9 of a series of 2-fold dilutions of the antibiotic or plus 5 mM metabolite. Compared with wells containing the antibiotic only, these wells containing 5 mM metabolite were used to evaluate the effect of the metabolite on bacterial susceptibility to antibiotic. The mixtures were incubated at 37°C for 8 h. MKC was defined as the lowest antibiotic concentration point that killed visible bacteria.

### Antibiotic bactericidal assay

Antibiotic bactericidal assay was performed as previously described (Jiang et al., 2020). A single colony was propagated in 50 ml of LB and cultured for 16 h at 37 °C in a shaker. The sample was collected by centrifugation at 8,000 rpm for 3 min and washed three times with saline solution. Then bacteria were resuspended in M9 to an optical density at 600 nm of 0.2 and diluted at 1:100. A metabolite and/or an antibiotic were added and incubated at 37°C and 200 rpm for 6 h. Aliquot of 100 μL samples were 10-fold serially diluted and aliquot of 5 μL of each dilution was spotted onto the LB agar plates and cultured at 37 °C for 11 h to determine CFU of bacteria. The percent survival was determined by dividing the CFU of the treated group by the CFU obtained from the control group.

### MBC determination

Following the determination of the MKC test result, aliquot of 100 μL mixture from a well of 96-well microtiter polystyrene tray was diluted 1:10-fold. Aliquot of 5 μL of each dilution was spotted onto the LB agar plates and cultured at 37°C for 11 h to determine the CFU of bacteria. MBC was defined as the lowest antibiotic concentration that killed bacteria to a CFU of less than 10,000 per well.

## Results

### Comparison on Viability, MIC, and MKC Between LB and M9 Medium

**FIGURE 1:**
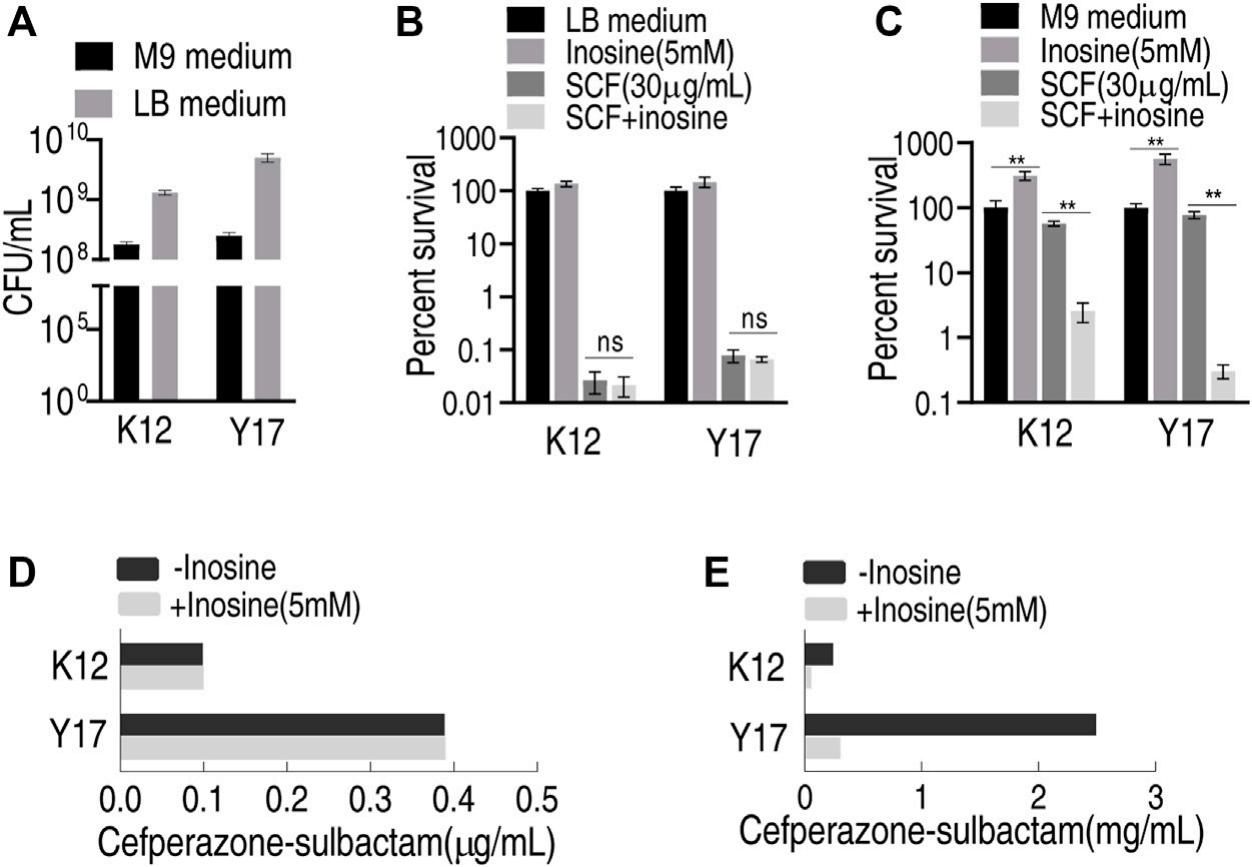
Effect of inosine in different medium **(A)** Viability of K12 and Y17 in LB medium and M9 medium at 37°C for 6 h **(B)** Percent survival of K12 and Y17 in LB medium with and without 5 mM inosine plus SCF **(C)** Percent survival of K12 and Y17 in M9 medium with and without 5 mM inosine plus SCF **(D)** MIC test of K12 and Y17 in LB medium with and without 5 mM inosine plus SCF **(E)** MKC test of K12 and Y17 in M9 medium with and without 5 mM inosine plus SCF. Results are displayed as mean ± SEM and three biological repeats are performed. Significant differences are identified *p < 0.05, **p < 0.01.

*E. coli* K12 and Y17 are antibiotic-sensitive and clinically multidrug-resistant *E. coli* strains, respectively. Both plate-counting and MIC tests were used to test whether inosine potentiated SCF to kill the two bacterial strains in LB and M9 medium. Different results were detected by the plate-counting method between LB and M9 medium. Specifically, bacteria grew faster and was more sensitive to SCF in the LB medium than the M9 medium under the same inoculation amount (Figures 1A–C). However, exogenous inosine distinctly promoted bacterial growth in the M9 medium compared with that in the LB medium. Consistently, viability of the two strains was similar in the LB medium plus SCF with and without inosine (Figure 1B), while lower survival of both strains was detected in the M9 medium with inosine plus SCF than without (Figure 1C). Similarly, the same MIC to SCF of the two strains was determined in the LB medium with and without inosine plus SCF (Figure 1D), but lower MKC was measured in the M9 medium with than without inosine (Figure 1E). These results indicate that the investigation on metabolite-enabled killing of *E. coli* by SCF should be performed in M9 medium.

### Optimization for Bacterial Number and Incubation Time in MKC Test

**FIGURE 2:**
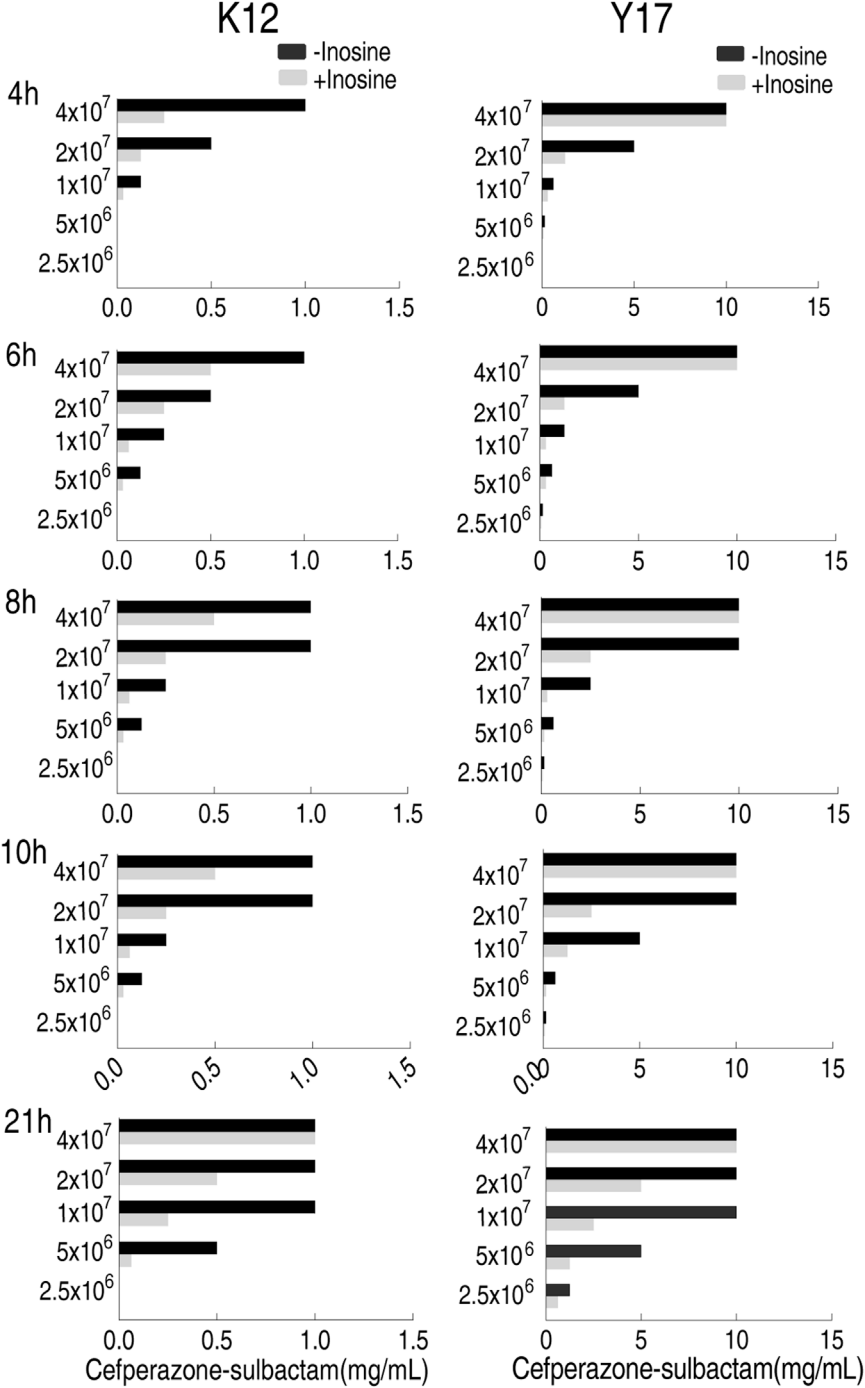
MKC of K12 and Y17 in five bacterial doses and five incubation time by SCF.

To optimize bacterial number in the MKC test, five bacterial doses, 2.5 × 10^6^, 5 × 10^6^, 1 × 10^7^, 2 × 10^7^, and 4 × 10^7^ colony forming unit (CFU), were adopted. Meanwhile, 5 incubation time with 4–21 h was used to optimize the incubation period. Inosine promoted SCF-mediated killing by eight MKC as the top efficacy. Comparatively, 1 × 10^7^ and 2 × 10^7^ CFU were the optimized numbers, while 8 h was the optimized incubation time. Specifically, four MKC was detected in medium with 5 × 10^6^ CFU at 6, 8, 10, and 21 h, with 1 × 10^7^ CFU at 4, 6, 8, 10, and 21 h, with 2 × 10^7^ CFU at 4, 8, and 10 h, with 4 × 10^7^ CFU at 4 h for K12; with 5 × 10^6^ CFU at 8, 10, and 21 h, with 1 × 10^7^ CFU at 6, 10, and 21 h, with 2 × 10^7^ CFU at 4, 6, 8, and 10 h for Y17. Notably, eight MKC was only detected in medium with 1 × 10^7^ CFU at 8 h for Y17. However, no difference was detected in medium with 2.5 × 10^6^ CFU at these time points (Figure 2). These results support the 1 × 10^7^ CFU and 8 h incubation time as optimizing conditions for the following MKC test.

### Optimization for Ionic Strength in MKC Test

**FIGURE 3:**
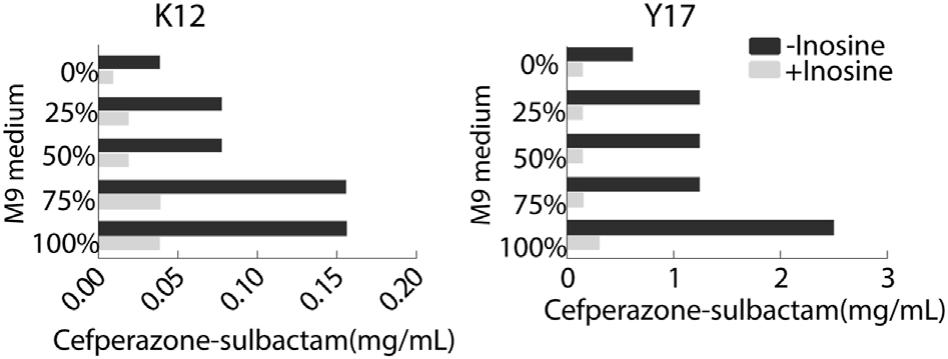
MKC of K12 and Y17 in the presence or absence of 5 mM inosine and different ionic strength by SCF.

To optimize ionic strength in the MKC test, the M9 medium was not diluted or diluted into 75, 50, 25% using saline solution or only saline solution, designed as 100, 75, 50, 25, and 0% M9, respectively. MKC was elevated with the increasing ironic strength in medium with or without 5 mM inosine. However, the inosine potentiated the SCF-mediated killing efficacy similarly (Figure 3). Notably, clear bacterial pellets dropped at the bottom of the plates in 100% M9. These results support 100% M9 as an optimizing condition for the following MKC test.

### Optimization for Inosine Concentration in MKC Test

**FIGURE 4:**
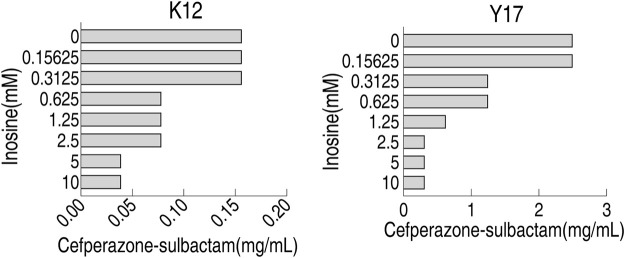
MKC of K12 and Y17 in different inosine concentration by SCF.

To optimize inosine concentration in MKC test, 0.15625, 0.3125, 0.625, 1.25, 2.5, 5, 10 mM inosine were used. Compared with control with 0 mM inosine, 0.15625 and 0.3125 mM inosine did not affect MKC, 0.625–2.5 mM inosine, and 5–10 mM inosine reduced MKC by 2 and 4-fold, respectively, for K12; 0.15625 mM inosine did not affect MKC, 0.3125 and 0.625 mM inosine, 1.25 mM inosine, and 2.5–10 mM inosine reduced MKC by 2, 4 and 8-fold, respectively, for Y17 (Figure 4). These results support the use of 5 mM inosine for the following MKC test.

### Test for Antibiotic Classes in MKC Test

**FIGURE 5:**
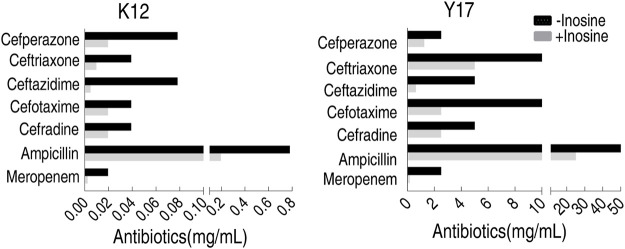
MKC of K12 and Y17 in the presence or absence of 5 mM inosine by seven antibiotics.

To test antibiotic classes used in the MKC test, cefoperazone, ceftriaxone, ceftazidime, cefotaxime, cefradine, ampicillin, and meropenem were used. Among them, cefoperazone, ceftriaxone, ceftazidime, cefotaxime, and cefradine belong to cephalosporins, while ampicillin and meropenem are classified as penicillins and carbapenems, respectively. Inosine potentiated these antibiotics to decrease the MKC of K12 and Y17, where the best efficacy was detected in ceftazidime for K12 and Y17 (Figure 5). These results indicate that the developed MKC test can be used to evaluate metabolites that promote these classes of antibiotics-mediated killing efficacy.

### Feasibility of the MKC Test for Different Metabolites to Clinically Multidrug-Resistant *E. coli* Strains

**FIGURE 6:**
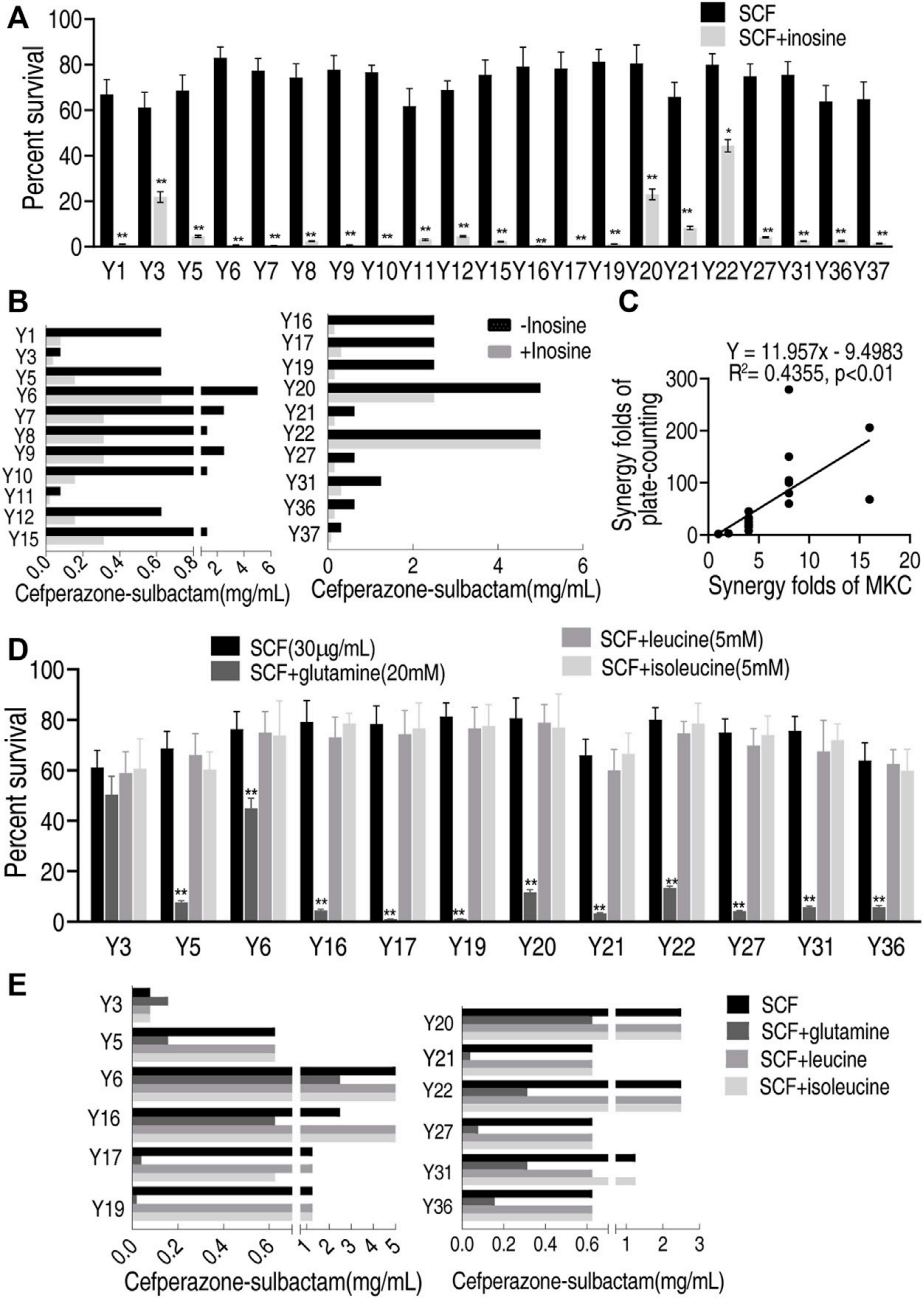
Effect of different metabolites on clinically multidrug-resistant *E. coli* strains by MKC test in M9 medium **(A)** Percent survival of 21 clinically isolated *E. coli* strains in the presence or absence of inosine in M9 medium by SCF **(B)** MKC test of 21 clinically isolated *E. coli* strains in the presence or absence of inosine in M9 medium by SCF **(C)** Correlation analysis between MKC test and plate-counting method **(D)** Percent survival of 12 clinically isolated *E. coli* strains in the presence or absence of glutamine, leucine, isoleucine respectively in M9 medium by SCF **(E)** MKC of 12 clinically isolated *E. coli* strains in the presence or absence of glutamine, leucine, isoleucine respectively in M9 medium by SCF. Results are displayed as mean ± SEM and three biological repeats are performed. Significant differences are identified *p < 0.05, **p < 0.01.

To test the feasibility of the MKC test for different metabolites, more clinically isolated *E. coli* strains and metabolites were used. Exogenous inosine promoted SCF to kill all of the 21 strains by plate-counting test, with viability decreasing from 1.8 to 279-fold (Figure 6A), while the promotion was detected in 18 out of 21 strains with MKC-fold from 4 to 16 but not the other three (Figure 6B). There was a positive correlation between the plate-counting test and MKC test (Figure 6C). These results indicate that the developed MKC test is feasible for these clinically isolated strains. On the other hand, three more metabolites, glutamine, leucine, and isoleucine, were utilized to test the feasibility of metabolites. The plate-counting test showed that glutamine potentiated SCF to kill 11 out of the 12 clinical *E. coli* strains used but leucine and isoleucine did not (Figure 6D). Equally, the MKC test demonstrated that glutamine had the potentiation that the plate-counting test did, whereas leucine and isoleucine did not (Figure 6E). These results indicate that the developed MKC test is feasible for the screening of metabolites that potentiate antibiotic-mediated killing. Therefore, the method is suitable for detection of a scale of clinical strains and different metabolites that promote antibiotics-mediated killing efficacy.

### Feasibility of the MKC Test for Clinically Multidrug-Resistant Strains by Using Inosine

**FIGURE 7:**
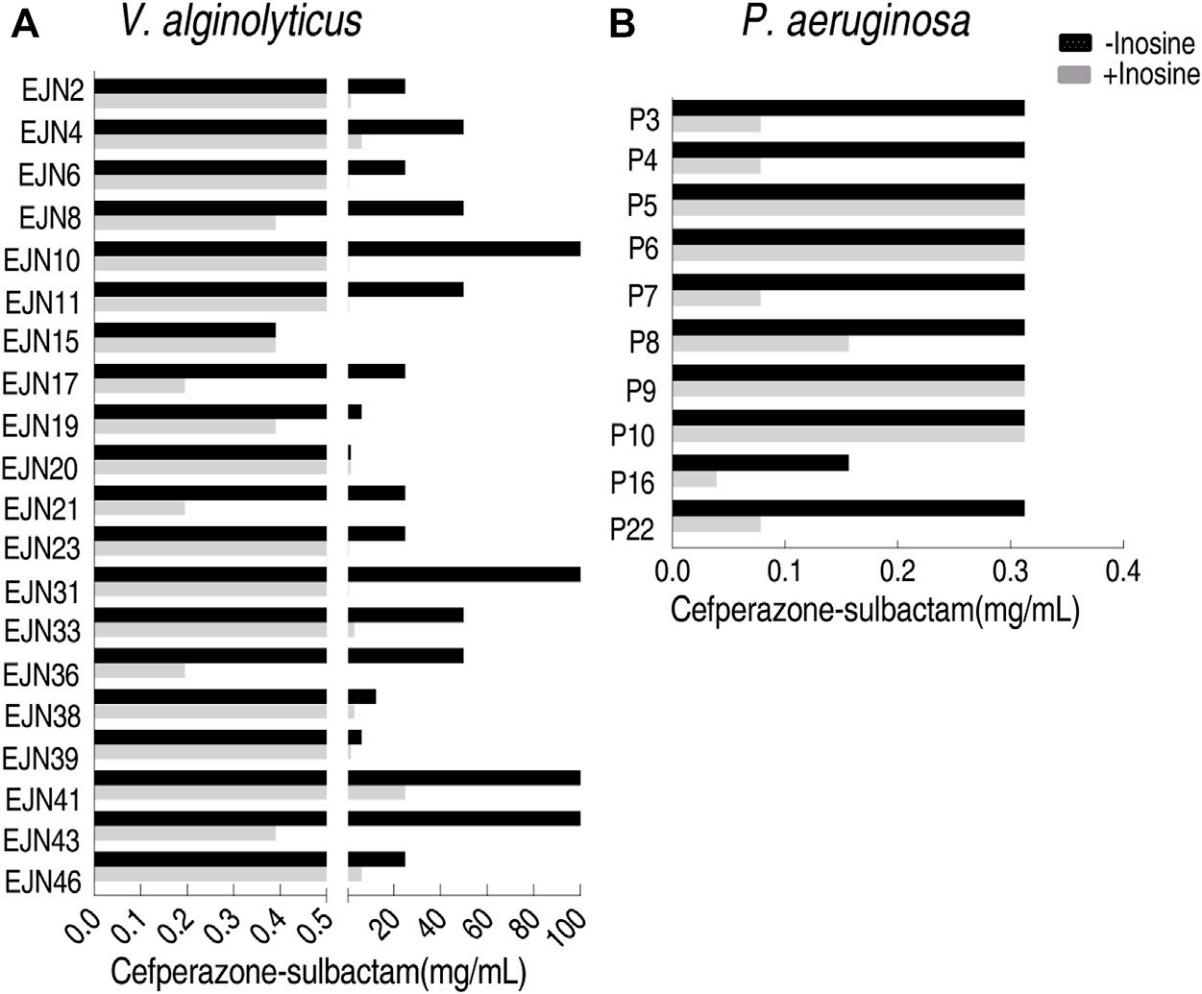
MKC test for multidrug-resistant strains by using inosine **(A)** MKC test of 20 aquatic product source *V. alginolyticus* strains in M9 medium with and without 5 mM inosine plus SCF **(B)** MKC test of 10 clinically isolated *P. aeruginosa* strains in M9 medium with and without 5 mM inosine plus SCF.

To test the feasibility of the MKC test for naturally-evolutionary multidrug-resistant pathogens, besides the above *E. coli* strains, *V. alginolyticus* and *P. aeruginosa* strains were also tested. Among 20 *V. alginolyticus* strains, 90% (18) exhibited 4–256 fold of reduction of MKC in the presence of inosine (Figure 7A), while the other two displayed the same MKC. The MKC test also showed that inosine reduces 50% (5/10) *P. aeruginosa* by four MKC (Figure 7B). Therefore, the method is suitable for detection of various isolated pathogens.

### Feasibility of the MKC Test to the Following MBC Test

**FIGURE 8:**
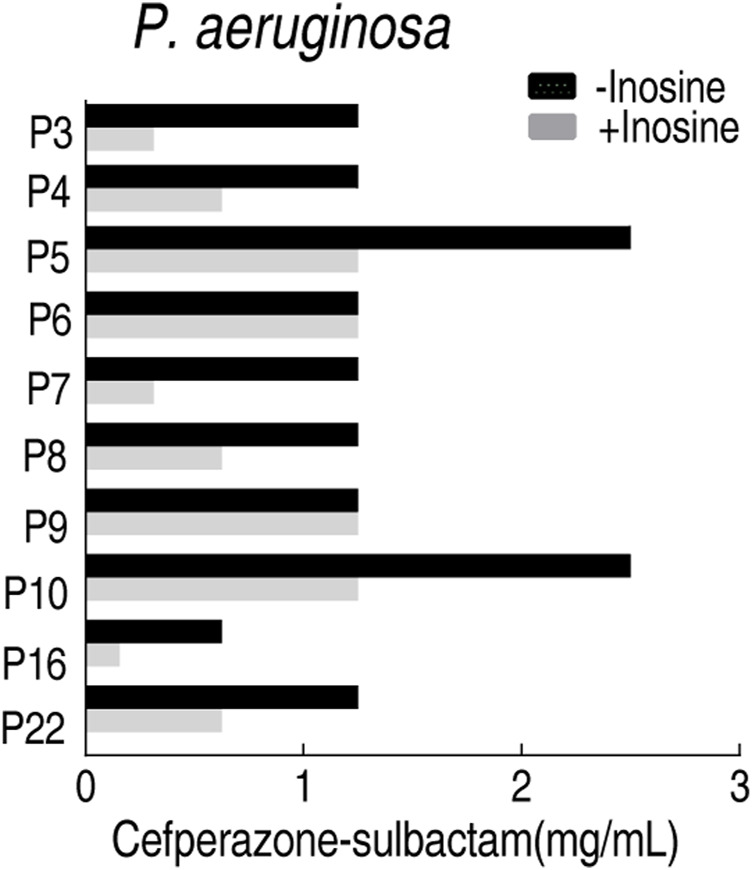
MBC test of 10 clinically isolated *P. aeruginosa* strains in M9 medium with and without 5 mM inosine plus SCF.

MBC is an important index to measure the antibacterial activity of antibiotics, which is usually followed by MIC. To test the feasibility of the MKC test for the following MBC test for clinically multidrug-resistant strains, the MBC test was followed by the above MKC test of *P. aeruginosa*. MBC values were obtained in the presence or absence of inosine. A lower MBC value was detected in the presence of inosine than absence, which is similar to the measurement of MKC in the same conditions (Figure 8). These results support the conclusion that the MBC test can be followed by the MKC test.

## Discussion

Reprogramming metabolomics is a recently developed approach to restore the killing efficacy of the currently used antibiotics (Peng et al., 2015a). To do this, the identification of metabolites that promote antibiotic-mediated killing is especially key. However, the currently used plate-counting method is not inappropriate as a scaleable screening method. Therefore, the present study tries to develop an extensive screening method for identification of the potentiated compounds including metabolites. To our knowledge, this is the first time that the MKC data of SCF with and without inosine in M9 medium have been reported. The possible mechanisms are attributed to the role of inosine as a regulator to CpxA/R system (Zhao et al., 2021).

A microtitre plate dilution method that measures MIC can be performed in full with samples to determine bacterial antibiotic resistance (Wiegand et al., 2008; Hall et al., 2011), but the method is not suitable to evaluate the potentiation caused by compounds including metabolites. This is because LB medium with rich nutrients contains many carbon sources. When a metabolite such as inosine is added, it may not be utilized by bacteria due to the existence of the other carbon sources. However, M9 medium contains minimal nutrients with only sodium acetate as a carbon source, and thereby the complemented inosine is easily used by bacteria. These conclusions are supported by the present findings that inosine enhances the bacterial growth in only M9 medium but not in LB medium and thereby inosine-enabled killing of bacteria by SCF was determined only in M9 medium. On the other hand, the nutritional difference between the LB medium and M9 medium allows bacteria to grow better in the LB medium than the M9 medium. Therefore, the MIC approach by inhibiting bacteria growth is not suitable to evaluate whether a metabolite has a potential effect of promoting antibiotic-mediated killing. It is necessary to develop a detection method that evaluates antibiotic-mediated killing efficacy instead of inhibitory efficacy.

Specifically, although MIC and MKC methods are used to test bacterial sensitivity to antibiotics, their purposes are different. MIC means the lowest concentration of antibiotics which inhibit the growth of bacterium and thereby leads to the maintenance or reduction of inoculum viability in rich medium (Andrews. 2001). However, MKC represents the lowest concentration of antibiotics which kill bacterium and thus causes the vanishing or reduction of inoculum viability in minimal medium. This causes two modifications of the MIC test to develop the MKC test. First, more bacterial cells are required in the MKC test than the MIC test. Second, M9 medium instead of LB medium is used to guarantee that bacterium efficiently utilizes the tested metabolites such as inosine in medium. The present study shows that the optimized cells are 1 × 10^7^ CFU - 2 × 10^7^ CFU in M9 medium for MKC test, which is different from 5 × 10^4^ CFU cells used for MIC in LB medium (Delgado-Valverde et al., 2020). Furthermore, the developed MKC test is examined by using several antibiotics, four metabolites, and 21 clinically isolated multidrug-resistant *E. coli*. Exogenous inosine can potentiate these antibiotics to kill *E. coli* in an MKC test, which is demonstrated as these antibiotics are bactericidal antibiotics, and as described above MKC represents the lowest concentration of antibiotics which kill bacterium. Meanwhile, the potentiation of four metabolites, inosine, glutamine, leucine, and isoleucine to SCF is evaluated in clinically isolated *E. coli* strains. The MKC test is proportional to a plate-counting test. These results indicate that a microtitre plate dilution method for MKC is developed to evaluate inosine-enabled killing of bacteria by antibiotics, which has benefits in screening compounds that potentiate antibiotic-mediated killing efficacy.

MBC is the minimum concentration of an antimicrobial drug that is bactericidal, while MKC is defined as the minimum killing concentration. Both are designated to measure the killing efficacy. MBC is identified by determining the lowest concentration of antibacterial agent that reduces more than 99.9% of the viability of the initial bacterial inoculum by plate counting after MIC. MKC is defined by identifying the lowest concentration of antibacterial agent that causes the vanishing or reduction of the initial inoculum viability directly. Therefore, compared with MBC, MKC is simple, economic, convenient, and time-saving.

Comparatively, the developed MKC is convenient, economic, accuracy, and suitable for detection of a scale of samples. It is suggested that samples should be examined using a plate-counting test when their MKC value ≤ 2. In addition, bacterial number is crucial in the developed MKC test, which may be related to bacterial species. Therefore, optimization of bacterial number is especially important in testing other bacterial pathogens using the developed MKC test.

## Conclusioin

The present study develops the MKC test to evaluate metabolites-enabled killing of clinically isolated *E. coli* by bactericidal antibiotics. The method is convenient, rapid, and economic, especially for a large scale of samples. These results suggest that a microtitre plate dilution method can be modulated to develop an MKC test to identify compounds that promote antibiotics to kill bacteria.

## Data Availability

The original contributions presented in the study are included in the article/supplementary material, further inquiries can be directed to the corresponding author.
